# Did the CP Audits Promote the Enterprises’ CP? A Case Study in Beijing

**DOI:** 10.1100/tsw.2002.143

**Published:** 2002-03-09

**Authors:** Gang Yu, Jun Huang, Qing Chen

**Affiliations:** Department of Environmental Science and Engineering, Tsinghua University, Beijing, 100084, China

**Keywords:** cleaner production, CP audits, enterprise, case study

## Abstract

Seven enterprises that have had recent Cleaner Production (CP) audits in Beijing were interviewed to identify whether these enterprises implemented the audit recommendations. If enterprises did implement the recommendations, their reasons and the results were analyzed. Finally, some suggestions on how to promote enterprise-wide CP were given.

## INTRODUCTION

Cleaner Production (CP) refers to the use of technology, processes, and management systems to avoid or minimize the creation of pollution. This is in contrast to "end of pipe" approaches to pollution prevention that focus on treating effluents and emissions after production. CP is thus an efficient use of resources producing the same, better, or more, with less raw materials, energy, and impact on the environment. CP concepts have been successfully introduced in many companies all over the world[1].

During the period 1993–1995, CP audits were conducted in 29 Chinese enterprises, supported by the World Bank and the United Nations Environment Program (UNEP), in cooperation with the National Environmental Protection Agency (NEPA) of China[2]. These were the first CP audits in China and then some institutions and companies did thousands of CP audits since 1993. This paper attempts to identify what role the CP audits played in the promotion of CP in China. A feasible approach to obtain reliable information was to visit typical enterprises belonging to different industrial sectors and communicate with people who had experience in CP practice. In April and May 2000, we visited seven enterprises in Beijing that have had CP audits to collect basic data on CP. As a result, we identified the common points among these enterprises, found out the key problems, and suggested corresponding solutions.

## METHODOLOGY

In order to evaluate the role of CP audits for promoting CP, we should know the current situation of CP, the management and operational structure of enterprises, and the barriers to implementing CP from the perspective of an enterprise's staff.

The ownership of an enterprise is very important in CP activities. State-owned enterprises (SOE) comprise a significant percentage of all industries in China. They tend to be large- and medium-scale industries, and are urban based. SOEs are defined as "industrial enterprises where the means of production or income is owned by the state"[3]. Representative sectors of SOEs include chemicals, electronics, pulp and paper, iron and steel, and other large, heavy industry. Among the seven selected enterprises in Beijing we visited, six were SOEs and four belonged to the chemical industry (Table 1).

**TABLE 1**
**The Ownership and Sector of the Selected Enterprises Visited**

| Enterprise No. | Sector | Type |
|---|---|---|
| Enterprise 1 | Metallurgy Industry | SOE |
| Enterprise 2 | Chemical Industry | SOE |
| Enterprise 3 | Chemical Industry | SOE |
| Enterprise 4 | Chemical Industry | SOE |
| Enterprise 5 | Chemical Industry | SOE |
| Enterprise 6 | Pharmaceutical Industry | SOE |
| Enterprise 7 | Electronic Industry | JV[a] |

[a] Joint-venture enterprise

A questionnaire was prepared for these enterprises, which contained seven main parts: general data, end-of-pipe environment, past CP audit activities, past CP demonstration projects, present CP situation, future CP plans, and suggestions.

We adopted two styles to collect CP information during our visits. One was to talk with one or two relevant managers or engineers; the other was to hold a discussion meeting attended by managers, technical staff, and operators. The latter approach was applied to all large-scale SOEs, such as Enterprise 1 and Enterprise 2.

## RESULTS AND DISCUSSION

### Current Situation of CP in Beijing: Becoming Better

CP is a new concept for many people in China, but that does not mean it is a wholly strange idea. In fact, many large-scale enterprises have done a lot in this field since 1993. Perhaps an important factor in CP's popularity is that Beijing is the capital of China, and both the central government and Beijing city authorities have paid more attention to pollution control. Since 1993, several international cooperative projects have been completed in China, such as the World Bank B-4 project, the Sino-Canada cooperative project, the Sino-Norway cooperative project, and others. Many large-scale enterprises have taken part in these projects. A number of CP audits have been completed and hundreds of people have received the special training. The related information is summarized in Table 2.

**TABLE 2**
**Some CP Information in Seven Visited Enterprises**

| Enterprise No. | CP Audits (Items) | Fund Input (RMB) | CP-Trained Staff (# of People) |
|---|---|---|---|
| 1 | 80 | 193,525,200 | 186 |
| 2 | 26 | 510,000,000 | Over 180 |
| 3 | 7 | >2,110,000 | ~200 |
| 4 | 3 | 420,000 | Not available[a] |
| 5 | 1 | 1,450,000 | Not available[a] |
| 6 | 5 | 1,840,000 | Over 100 |
| 7 | 1 | 1,400,000 | ~10 |

[a]Not available because of the retirement of some relevant people or staff changes.

As environmental problems became more and more serious, enterprises faced pressure both from the central government in Beijing and from the public as their environmental awareness was awakened. For some enterprises belonging to ministries and commissions, the higher pressure forced them to take measures to do CP audits.

In our list, Enterprises 1 through 4 joined all or part of the above CP projects. For example, Enterprise 2 claimed that more than 26 CP audits were completed in recent years, and more than 180 persons attended courses about CP. The company said that they not only took part in the standard courses, but also organized some special courses of their own.

## The CP Audit from an Enterprise's Perspective: A Good Thing, But Not So Easy to Implement

Through 8 years of experience, most enterprises have realized the benefits of CP. They now regard CP as a win-win strategy for an enterprise's development. On the one hand, enterprises reduced the pollutants they would have produced everyday in the past, which helps protect the environment. On the other hand, enterprises lowered their cost for products because they succeeded in reducing wasted raw materials and energy. The concept of CP has become increasingly familiar to people in the enterprises, both in SOEs and joint-venture (JV) enterprises.

The CP concept has been more and more accepted in many enterprises, both for workers and managers. When they talked about CP audits, all the people we interviewed said it was a good thing. But they also pointed out some barriers or difficulties in completing the CP audit process. These are due to the government, the enterprise itself, the public, and other factors:

### *The Government: Lacking Encouraging Laws, Regulations, and Policy*

There are no formal CP laws or regulations yet, except a local regulation, which seems not so practicable in Taiyuan[4]. Because enterprises cannot see an immediate benefit coming from the government, such as a tax discount or award for good CP implementation, many prefer to delay the audits rather than do them on their own initiative.

### *The Enterprise: Lacking Proper Management Mechanism to Promote CP Audits*

Due to the relatively stiff management, those technical workers who have done voluntary CP audit tasks have not received any profit. This factor is especially serious in SOEs. Employees may do a CP audit voluntarily once, or even twice, but few would do it for the third or fourth time.

Another factor is that managers may not understand the benefits of CP as clearly as the technical staff in some enterprises. The will of the engineers might not be easily transferred to the manager, who makes the final decision regarding CP.

### The Financial Limit: A Problem Especially in Old Enterprises

Funding for CP is limited for many older enterprises. These enterprises often have heavy financial burdens, while their older equipment cannot produce as much as newer enterprises. This may create a barrier to persuading others when the decision maker tries to spend the large funds necessary to complete the CP audit.

Loans for larger enterprises is not enough of an attraction, but still interested small enterprises, because larger enterprises can offer by themselves if only one choice — loaning from government or other ways, no matter high or low loan.

### The Technique Limit: Three Main Parts

First, the methodology of a CP audit may have some technical shortcomings itself. For example, the vice-head of the Safety and Environmental Protection Department of Enterprise 2 thought the benefit was not so apparent according to the methodology of the CP audit. The contrast in results is sometimes hidden on paper, and might not be persuasive enough for the leaders of the company.

Second, there are not enough CP techniques for specific industrial sectors that fit the current situation in China. For example, Enterprise 1 is looking for the proper solution to solve its emission during the rolling process, but Enterprises 4 and 5 are looking for good processes to control the water leakage in water nets.

Third, measurement equipment is still a problem for environmental management. For example, Enterprise 1 invested a lot of money in its seven subenterprises to reduce pollution and the cost for raw materials. They eventually succeeded in shortening their daily wastewater production, but they must pay as much as before because there is no proper measurement equipment for the complex wastewater-pipe net.

### The Information Limit: A Problem Especially in Knowing Advanced Technique

The main approaches to get information about CP are through newspapers, books, periodicals, and training courses held by the government. Some enterprises get information from networks; however, in China, there is little CP information in the network. There are few techniques for sharing CP information across similar sectors.

## Comparison Between SOE and JV Enterprise in CP Audit

### Difference in the Mode of Management

Enterprise 7 (a JV enterprise) has a serious mode of management. They seek not only profits, but also environmental benefits that have been emphasized since the company was established in China. They also have rigorous duty system, which is to try their best to do everything well. This management style is rooted in their parent company, so they accept CP relatively easily compared to SOEs. For example, Enterprise 7 has a back-check from its parent company every year, including the mode of management, technique, and environmental protection.

SOEs also try to pursue both profits and environmental benefits, but because of their long histories and differences in their modes of management, they meet a lot of difficulty when implementing CP audits. SOEs themselves lack a back-check for their work.

Another factor, which may be a barrier to implementing CP in SOEs, is the lack of funding, which results in that the managers of SOEs would like to pursue current profits. They have no choice but to promote production under existing conditions. They know that CP is a more efficient approach, increasing profits from the beginning to the end of the whole process of production, but it requires a lot of initial investment. Some SOEs also lack rewards and punishment for CP implementation, and cannot make their staff do a CP audit.

### Difference in Technique Investing

Some SOEs have a long history, and their techniques are often antiquated. In a way, implementing CP is more like a technical improvement. The managers do not want to not spend much money on environmental protection projects, so little money is used in implementing CP audits.

### Difference in Getting CP Information

Enterprise 7's good contact with its parent company makes it easier to get more information about CP. This is an easy and efficient way to get CP information.

SOEs get their information mainly through news, periodicals, and training courses held by the government. They lack specific CP technical information on their sectors and do not know where to seek help.

## SUGGESTIONS

The following steps can be taken to promote CP in enterprises:

1. The government should adopt encouraging laws or regulations for some special industrial sectors that are heavy polluters. In the meantime, it is better to encourage policies that promote the CP audit. If possible, a CP-labeling project and preferential duties may be good ways to stimulate enterprise to complete more CP audits.
2. Enterprises should reform their stiff management mechanism in order to make staff more actively amenable to the audit task. The decision-makers should become more aware of the CP concept, and more open to the appeals for CP from their technical staff.
3. Financial organizations should provide financial aid and help enterprises to get the proper techniques and build up an effective supervisor program that ensures the money is being used reasonably.
4. The relevant departments and organizations should develop good CP techniques for enterprises. It is better to provide more chances for enterprises and developing organizations to exchange ideas and information about CP, such as holding special meetings and building up the CP website. In the meantime, affected organizations should begin an educational campaign to improve the public awareness about cleaner production.
